# Correction to “Role‐Specific Performance Profiles of Professional Cyclists in a Grand Tour”

**DOI:** 10.1002/ejsc.70235

**Published:** 2026-08-01

Sánchez‐Jiménez, J. L., A. Javaloyes, D. Barranco, I. Peña‐González, M. Moya‐Ramón, and M. Mateo‐March. 2026. “Role‐Specific Performance Profiles of Professional Cyclists in a Grand Tour.” *European Journal of Sport Science* no. 6 (June): e70170. https://doi.org/10.1002/ejsc.70170.

There was a typographical error in the author name “David Barranco”. It should have been “David Barranco‐Gil” instead. The error has now been corrected in the article.

We apologize for this error.

